# Septo-optic dysplasia plus: a case report

**DOI:** 10.1186/1756-0500-7-191

**Published:** 2014-03-28

**Authors:** Lepsa Zoric, Simon Nikolic, Milan Stojcic, Dragana Zoric, Sinisa Jakovljevic

**Affiliations:** 1 Ophthalmology Department, Faculty of Medicine, University of Pristina, Settlement Kosovska Mitrovica, Anri Dinana bb, 38200 Kosovska Mitrovica, Kosovo-Serbia; 2 Radiology Institute, Faculty of Medicine, University of Pristina, Settlement Kosovska Mitrovica, Anri Dinana bb, 38200 Kosovska Mitrovica, Kosovo-Serbia; 3 Railway Health Institute, Ophthalmology Department, Savska 23, 11000 Belgrade, Serbia; 4 Neuropsychiatry Department, Faculty of Medicine, University of Pristina, Settlement Kosovska Mitrovica, Anri Dinana bb, 38200 Kosovska Mitrovica, Kosovo-Serbia; 5 Gynecology Department, Health Center Zvecan, Kosovska Mitrovica, Kosovo-Serbia

**Keywords:** Septo-optic dysplasia, Optic hypoplasia, Polymicrogyria, Hypopituitarism, Oculomotor palsy

## Abstract

**Background:**

Septo-optic dysplasia, also referred to as de Morsier syndrome, is a congenital condition characterized by classic triad features: midline brain abnormalities, optic nerve hypoplasia and pituitary endocrine dysfunction. Sometimes, other various malformations appear within syndrome.

**Case presentation:**

An 11 and 1/2-year-old Caucasian Southeast European female patient with earlier established diagnoses of growth hormone deficiency, diabetes insipidus, seizures, mental retardation, optic nerve atrophy and right ptosis, was directed to us for consultative examination.

The girl of short stature and low weight for her age had bilateral optic nerve hypoplasia, poor vision, nystagmus and right eye oculomotor palsy. Electroencephalogram revealed epileptic changes. Magnetic resonance imaging showed an empty sella syndrome, partial hypoplasia of corpus callosum, cavum of pellucid septum and diffuse polymicrogyria of the left temporal lobe. We found all elements of septo-optic dysplasia plus syndrome with right oculomotor nerve involvement.

**Conclusion:**

By earlier findings and evaluation, we established a diagnosis of septo-optic dysplasia plus. The case confirms the existence of various malformations within the syndrome and the need for the cooperation of several specialists in the diagnosis and treatment of children with the syndrome.

## Background

Optic nerve hypoplasia is the most common congenital optic nerve anomaly. It has variable fundoscopic and functional manifestations. As these manifestations can be from minimal to marked, optic nerve hypoplasia is not always a recognized ophthalmologic condition.

The term septo-optic dysplasia (SOD) was coined in 1956 by De Morsier, who pointed out the association of optic nerve hypoplasia with an absence of the septum pellucidum. In addition, in 1970, Hoyt and colleagues reported the association of SOD with pituitary dwarfism.

SOD is a clinically heterogeneous disorder characterized by the classical triad of optic nerve hypoplasia, pituitary hormone abnormalities, and midline brain defects. Its incidence is 1 in 10,000 live births and it has equal prevalence in males and females. Clinical diagnosis requires the presence of at least two characteristics and can be confirmed by ophthalmological examination, magnetic resonance imaging (MRI), and pituitary hormone analyses. The most common endocrine abnormality is growth hormone deficiency, followed by gonadotropin deficiency, but it can also be caused by decreased secretion of other hormones (corticotropin, antidiuretic hormone, and thyroid stimulating hormone (TSH)). The occurrence of a wide variety of developmental ocular, brain, and other abnormalities has been recognized (corpus callosum dysgenesis, schizencephaly, Duane syndrome, hemi-facial atrophy and midfacial anomalies, blepharophimosis, microphthalmos, anophthalmia, strabismus, olfactory tract hypoplasia, precocious and late puberty, microphallus, autism, and cardiac and skeletal malformation) [1-5].

The causes of this early brain midline dysembryogenesis are unknown, but the most frequently suggested etiologies relate to embryonic vascular insult, usually in the 6^th^–7^th^ week of embryogenesis, bleeding during the first trimester of pregnancy, primiparity and young maternal age [6], and maternal alcoholism or drug abuse during pregnancy [5]. Familial cases point to a mutation in a developmental gene (*HESX1*, *SOX2*, *SOX3*) [6].

In this report we describe the association of SOD with other malformations.

## Case presentation

An 11-and-a-half-year-old female Caucasian patient from Southeast Europe (Serbia), with previously diagnosed pituitary hormone deficiency, diabetes insipidus, optic nerve atrophy, seizures, and mental retardation was referred to us for examination.

She was the first child of young, healthy parents (her mother was 23 during the pregnancy and denied the use of alcohol or drugs). The pregnancy ended in the 36th week by cesarean section because of signs of fetal distress. Because of perinatal asphyxia and jaundice with indirect hyperbilirubinemia, the newborn spent one week in incubator and on phototherapy. During the first weeks of the girl’s life, her mother noticed that the baby was flaccid, did not awake spontaneously to feed, sucked weakly and vomited after every feeding, and did not attain normal growth and weight milestones. Neonatal convulsions appeared at the end of the first month. A diagnosis of central diabetes insipidus was established in the second month of life in a regional hospital, and she began therapy. Congenital right ptosis and bilateral nystagmus were also noted at this time. At the end of her first year, growth retardation was obvious and, after examination, growth hormone was prescribed, although irregularly applied. At the age of 5, she had a comitial crisis and antiepileptic therapy was introduced. Triiodthyronine (T3), thyroxine (T4), and thyroid-stimulating hormone (TSH) deficit was recognized when she was 7.

**Figure 1:**
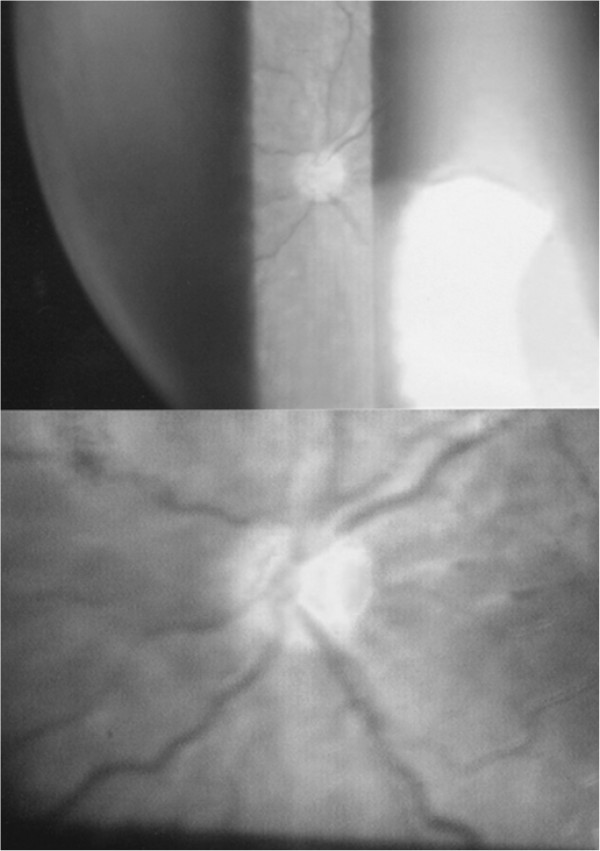
Optic nerve hypoplasia, right eye.

**Figure 2:**
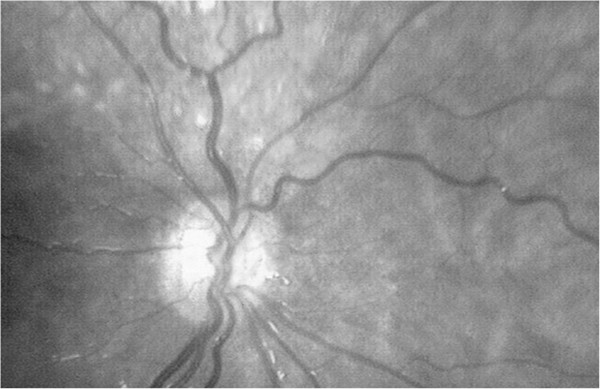
Left eye (small, pale optic disc with double ring sign).

When she came to us, she was 11-and-a-half-years old, her body weight was 20 kg, and her height was 115 cm. Her mental and cognitive functions were near normal range (Interim Hayes-Binet intelligence scale test score, 81). Laboratory hormone results were: growth hormone (GH or somatotropine STH) 1.23I ng/ml, follicle-stimulating hormone (FSH) 2.7 mIU/ml, luteinizing hormone (LH) = 2.052 mIU/ml, TSH = 0.97 mU/L, T4 = 116.9 nmol/L, cortisol 380 nmol/L, and adrenocorticotrophin hormone (ACTH) 17.8 pg/ml. She had visual acuity 0.1 on the right and 0.2 on the left, which could not be corrected (6/6 vision), bilateral optic nerve hypoplasia (Figures 1 and 2), bilateral marked horizontal nystagmus, and discrete anisocoria (left pupil wider by 1 mm) with a bilateral weak response to light stimuli.

Her right congenital ptosis was associated with ocular motility disturbances. Active palpebral elevation was impossible and her eyelid edge reached the pupillary border. Her eye was deviated downwards and laterally in the primary position.

Except as described, the findings on the other cranial nerves of the girl were normal. She was without sensory and motor deficits and cerebellar dysfunctions. Upon further investigation, an electroencephalogram revealed bilateral epileptic changes posteriorly with a maximum in the right superior temporal sulcus (TPO area).

**Figure 3:**
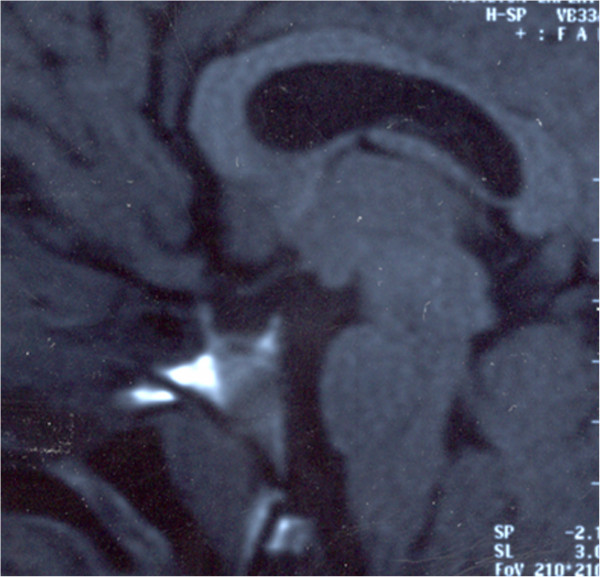
Sagital T1W magnetic resonance image shows a small optic chiasm, a hypoplastic pituitary stalk, an “empty” sella and a partial hypoplasia of corpus callosum.

**Figure 4:**
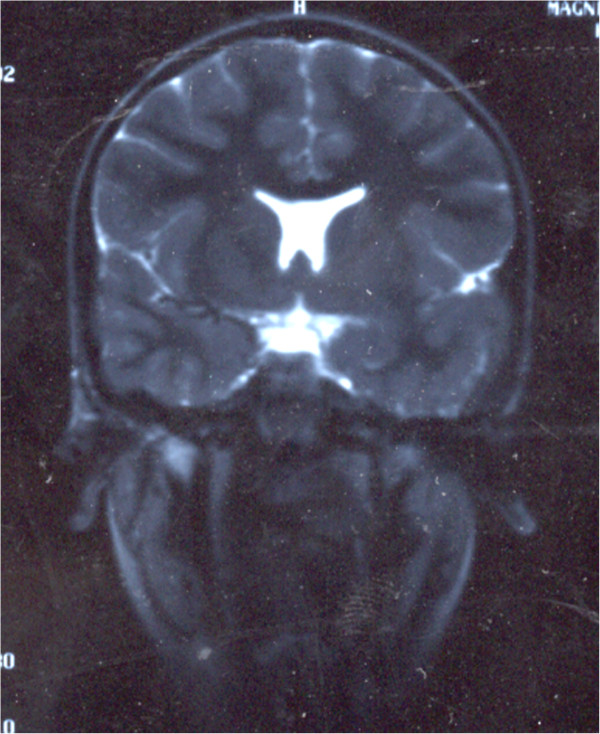
Coronal T2W magnetic resonance image shows the absence of the septum pellucidum, a flat roof of the lateral ventricles, a “point down” appearance to the inferior parts of the frontal horns, the loss of normal gyral architecture of the left temporal lobe, thickened cortex with indistinct corticomedullary interface (polymicrogyria) and enlarged left Sylvian fissure.

MRI showed empty sella syndrome, partial hypoplasia of the corpus callosum (Figure 3), diffuse polymicrogyria of the left temporal lobe, and agenesis of the septum pellucidum (Figure 4).

The girl was directed to a pediatric endocrinologist to complete the evaluation and to the Strabismus Department for observation of the ocular muscles and correction of the ptosis. No reports were returned.

## Discussion

This case report describes a girl with fully expressed SOD and some accompanying findings. Besides SOD, the patient had diffuse polymicrogyria of the temporal lobe and disturbance of the levator palpebrae and extra-ocular muscles of the right eye. A marked nystagmus and the position of her right eye made it impossible to take adequate photos of the fundus of her right eye.

Schizencephaly is the most frequently recognized malformations of cortical organization within SOD. Other forms of cortical dysplasia in SOD, which exclude schyzencephaly, are already described and some of them are referred as ‘SOD-plus’ [7]. The cortical malformations are usually accompanied with psychomotor developmental delay and motor deficits. Because of phenotypic heterogeneity, not only related to the brain malformations and function, the term ‘SOD-complex’ was also proposed [8]. The cortical malformations are usually accompanied by psychomotor developmental delay and motor deficits. In our patient, no dysfunctions were obvious except for the epileptic changes. Previously noted mental retardation was explained as educational neglect, because the child did not attend any educational institution before the first test. Our recommendation in further was a school for visually handicapped children. An adequate rehabilitation program can play a crucial role in promoting neuropsychomotor development and delaying such an intervention may negatively influence this development. According to other reviews [9], patients with SOD-plus seemed to have a more favorable cognitive profile, but greater risk of major neurological involvement.

The possibility of diagnosing prenatal cerebral changes by three-dimensional ultrasound and fetal MRI has been reported in the literature. MRI is usually most helpful to visualize midline and other brain abnormalities in children with optic nerve hypoplasia [1].

Nearly two thirds of SOD patients have pituitary hormones deficiency. It is not always clear if the hypopituitarism is due to a primary pituitary dysfunction or is secondary to a hypothalamic dysfunction. Lack of corticotropin and/or antidiuretic hormone and poor thermoregulation can be a cause of sudden death during febrile illness [9-11]. Cholestatic jaundice and hypoglycemia, which are characteristic of children with SOD, are usually connected with ACTH and TSH deficit.

The optic nerve hypoplasia can be uni- or bilateral, but some level of poor vision and nystagmus are present in most cases of SOD. The “down and out” position of our patient’s right eye was typical of oculomotor nerve palsy. Partially preserved ocular movement may indicate more severe damage to the superior oculomotor nerve division or maldevelopment of the levator palpebrae and superior rectus muscles. The difference in pupillary size was not significant and assessment of the pupil response was hampered by bilateral optic nerve hypoplasia. Oculomotor dysfunction has been described in this syndrome [5,11,12], although it’s etiological association with other aspects of the syndrome is not clear.

## Conclusion

De Morsier syndrome is a spectrum of disorders; this case confirms this statement. Our patient presented bilateral optic nerve hypoplasia, ocular motility disturbances, midline brain defects with multiple pituitary hormone deficiency and left temporal lobe polimycrigyria, but in the absence of clear neurological deficit. Optic nerve hypoplasia and other ophthalmologic disorders may not be recognized during the first months of life, but should be borne in mind and investigated in such children. Early diagnosis might have preventive potential. A life-long multidisciplinary approach is crucial in the management of these patients to optimize their growth and development and to help them lead as normal a life as possible.

## Consent

Written informed consent was obtained from the patient’s guardian/parent/next of kin for the publication of this report and any accompanying images.

## Competing interests

The authors declare that they have no competing interests.

## Authors’ contributions

LZ diagnosed disease and wrote the manuscript. SN did MRI diagnostic. MS organized other tests. DZ helped with neurological aspect of the disease. SJ organized other consultations. All authors read and approved the final manuscript.
